# Ranolazine for Angina in Hypertrophic Cardiomyopathy

**DOI:** 10.1155/2018/5142572

**Published:** 2018-04-15

**Authors:** Akhtar Amin, Bernard Kim

**Affiliations:** ^1^Department of Internal Medicine, Rutgers New Jersey Medical School, Newark, NJ, USA; ^2^Department of Cardiology, Hackensack University Medical Center, Hackensack, NJ, USA

## Abstract

Ranolazine is an antianginal and works by inhibiting late-sodium current (*I*_NaL_). However, its use is limited mostly to patients with coronary artery disease. However, literature has shown its potential benefit in relieving angina in hypertrophic cardiomyopathy. Hereby, we discuss two cases where ranolazine led to improvement in angina refractory to beta-blockers. In conclusion, ranolazine can be considered as a potential antianginal drug in patients with hypertrophic cardiomyopathy refractory to beta-blockers.

## 1. Introduction

Ranolazine is a novel antianginal drug which was approved by FDA a decade ago [1]. At the time of its approval, the mechanism of action of ranolazine was unknown [1]. Furthermore, the antianginal effect of ranolazine was mostly studied in patients with coronary artery disease [2, 3].

In 2012, AHA/ACC published its guidelines for treatment of stable angina in patients with coronary artery disease, which included a class IIa recommendation stating ranolazine as a second-line therapeutic agent [4].

However, over the past decade, our understanding of ranolazine has developed significantly. It has been established that ranolazine acts a late sodium current (*I*_NaL_) inhibitor. Its role in decreasing diastolic myocardial wall tension and subsequently increasing the coronary blood flow during diastole has been studied [5]. Hypertrophic cardiomyopathy is a new clinical ground where ranolazine has shown promising results in terms of its antianginal effects [6].

We hereby discuss two cases of hypertrophic cardiomyopathy with angina refractory to beta-blockers, where ranolazine led to improvement in symptoms.

## 2. Cases

### 2.1. Case #1

A 31-year-old female, with a known diagnosis of hypertrophic cardiomyopathy, presented to the ED with intermittent chest pain despite being on metoprolol succinate 100 mg daily. A transthoracic echocardiogram (TTE) showed marked septal hypertrophy with a resting outflow gradient of 80 mmHg. After ruling out other possibilities, diagnosis of chronic stable angina was made. She was discharged after an increase in beta-blocker dosage and was followed up as an outpatient. The patient returned to the ED the next year with similar symptoms. Left heart catheterization showed normal coronaries, and the patient was started on ranolazine, which was titrated up to ranolazine 1000 mg BID as an outpatient. Symptoms improved significantly on combination of metoprolol and ranolazine.

### 2.2. Case #2

A 49-year-old male, with no past medical history, presented to the ED with exertional SOB and episode of lightheadedness. TTE showed severe left ventricular hypertrophy (LVH) with impingement of the septum into left ventricular outflow tract (LVOT) with significant LVOT obstruction and a potential intracavitary gradient >100 mmHg. Left cardiac catheterization showed normal coronaries. Thus, a diagnosis of hypertrophic cardiomyopathy (HCM) was made. He was discharged on metoprolol 25 mg TID. The patient was seen as an outpatient where he complained of anginal symptoms. Provided his heart rate was in low 60 s, he was started on ranolazine 500 mg BID, which was titrated up to 1000 mg BID on the subsequent visit. The patient reported significantly improved symptoms on the combination of metoprolol and ranolazine.

## 3. Discussion

Ranolazine is an effective antianginal therapy for patients with hypertrophic cardiomyopathy (HCM). HCM is a very commonly inherited cardiac disorder, with prevalence of 1 in 500 in general population [7]. Chest pain-like angina is a very frequent presenting symptom of HCM and has been postulated to be due to microvascular ischemia [7]. Beta-blockers and calcium channel blockers have been effective in managing angina in HCM. However, for patients with symptoms refractory to these medications, ranolazine is an effective therapy.

Coppini et al. [5] demonstrated an increased density of *I*_NaL_ in cardiac myocytes isolated from patients with HCM. *I*_NaL_ is a persistent slow influx of sodium ions throughout the action potential of cardiac myocyte. This persistent influx is due to the delayed inactivation of the sodium channel. An increase in intracellular sodium leads to increased intracellular calcium via decreased activity of energy-requiring Na-Ca exchanger (NCX), and in some cases, even via reverse flow through the NCX [8].

An increase in intracellular calcium level, in turn, leads to increased tension in the LV myocardial wall which contributes to diastolic dysfunction [8]. This worsening diastolic dysfunction increases left ventricular end-diastolic pressure (LVEDP) which, in turn, decreases the coronary flow gradient; and ultimately a decrease in the blood supply to the myocardium is observed.

Coppini et al. also demonstrated lengthening of action potential duration (APD) in cardiac myocytes of HCM patients, also indicatively increased intracytosolic calcium. They successfully reported the in vivo effect of ranolazine in shortening of APD.

This consolidates that ranolazine's function as a late sodium current (*I*_NaL_) inhibitor and prevents the increase in intracytosolic calcium, ultimately increasing the blood supply to the myocardium and relieving anginal symptoms.

A study by Gentry et al. [6] assessed the efficacy of ranolazine in relieving anginal symptoms refractory to maximum tolerated medical therapy in HCM. Among the patients that successfully completed the study, a significant reduction in anginal symptoms was reported with addition of ranolazine.

## 4. Conclusion

Although it requires further assessment by randomized-controlled trials, ranolazine can be considered as a possible antianginal therapy in HCM patients with symptoms refractory to beta-blockers or calcium channel blockers.
